# Supplementary material for: Exploring a blue-light-sensing transcription factor to double the peak productivity of oil in Nannochloropsis oceanica
Source: Nat Commun. 2022 Mar 29;13:1664. doi: 10.1038/s41467-022-29337-x (PMC8964759; doi:10.1038/s41467-022-29337-x)
Supplement: Supplementary file 1 — Supplementary Information [file 41467_2022_29337_MOESM1_ESM.pdf]

Supplementary Information

Exploring a blue-light-sensing transcription factor to double the peak productivity of oil in *Nannochloropsis oceanica*

Peng Zhang [1, 2, 3, †], Yi Xin [1, 2, 3, †], Yuehui He [1, 2, 3], Xianfeng Tang [3, 4], Chen Shen [1, 2, 3], Qintao Wang [1, 2, 3], Nana Lv [1, 2, 3], Yun Li [1, 2, 3], Qiang Hu [2, 5], Jian Xu [1, 2, 3, *]

[1] Single-Cell Center, CAS Key Laboratory of Biofuels, Shandong Key Laboratory of Energy Genetics and Shandong Energy Institute, Qingdao Institute of Bioenergy and Bioprocess Technology, Chinese Academy of Sciences, Qingdao, Shandong 266101, China

[2] Qingdao National Laboratory for Marine Science and Technology, Qingdao, Shandong 266237, China

[3] University of Chinese Academy of Sciences, Beijing 100049, China

[4] Qingdao Engineering Research Center of Biomass Resources and Environment, Qingdao Institute of Bioenergy and Bioprocess Technology, Chinese Academy of Sciences, Qingdao, Shandong 266101, China

[5] Institute for Advanced Study, Shenzhen University, Nanshan District, Shenzhen, Guangdong 518060, China

[†] These authors contributed equally to this article.

[*]Correspondence: Jian Xu (xujian@qibebt.ac.cn)

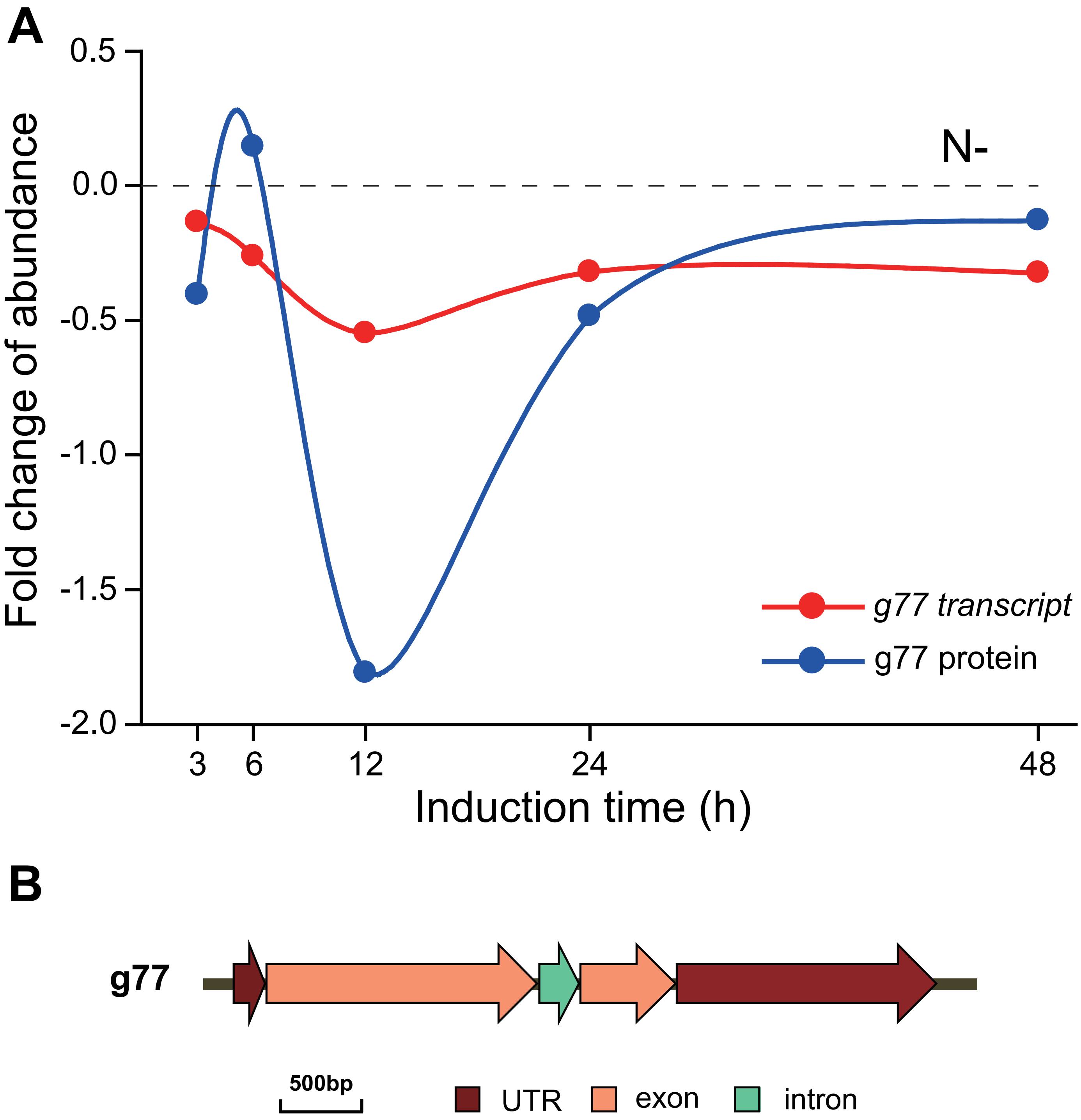

Supplementary Fig. 1. The gene expression pattern and gene structure of *Nannochloropsis oceanica scaffold00007.g77* (*NobZIP77*). (**A**) The temporal expression patterns of *g77* transcript and g77 protein are largely consistent under N- (note that at 6h, there is no significant fold change of g77 protein abundance between N+ and N-; p value = 0.41 [1]). Fold change of transcript abundance was calculated as log_2(FPKM(Tx, N-)/FPKM (Tx, N+)) (FPKM, the normalized abundance of transcript; Tx, time point). Fold change of protein abundance was calculated as (LFQ intensity (N-) - LFQ intensity (N+)). LFQ, label-free relative quantification. The transcriptome data [2] and the proteome data [1] were produced by distinct batches of experiments from two separate studies. (**B**) Gene structure. Orange, exon; green, intron; dark red; UTR, untranslated region. Arrow indicates the direction of transcription. The genomic DNA and cDNA sequences were both obtained by PCR amplification and sequencing. Source data are provided as a Source Data file.

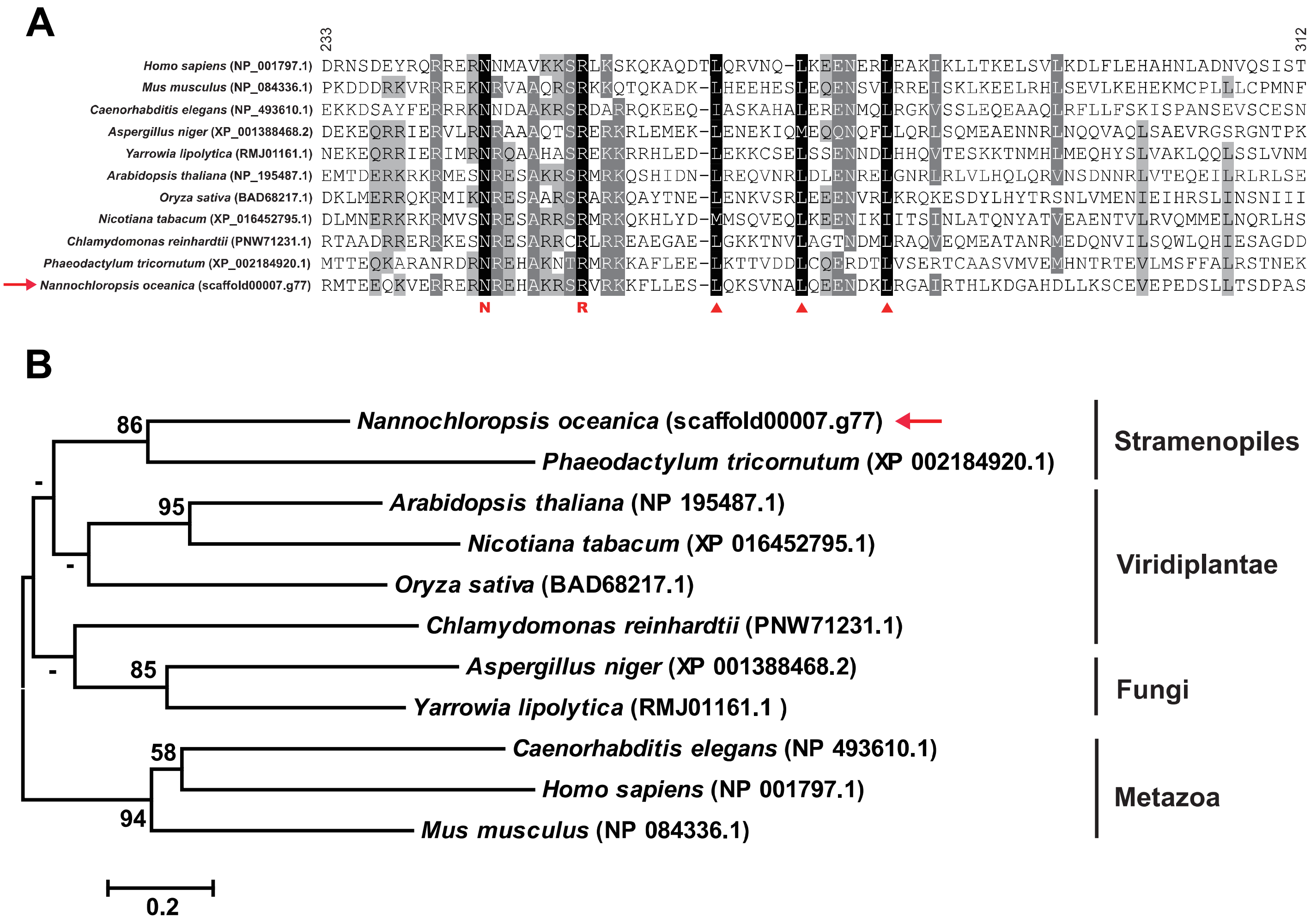

Supplementary Fig. 2. Phylogenetic analysis of NobZIP77. (**A**) Alignment of amino acid sequences among the bZIP domains from model species including animals, higher plants, fungi and microalgae. The bZIP domains were chosen from published proteins sequences that carry the domain. Notably, among the proteins shown, only g77 carries the LOV domain (which is related to blue light sensing). Accession numbers are shown. Bold residues below the sequences: conserved sites. Red triangles: putative leucine zipper. Red arrow: NobZIP77. Numbers above the sequences: amino acid position on NobZIP77. (**B**) Cladogram of selected bZIP-family protein sequences from higher plants, fungi, microalgae and animals. Neighbor-joining (NJ) was used for tree construction. Cladogram was plotted based on actual branch length. GenBank accession numbers are provided in brackets. Numbers beside the branch: bootstrap value for NJ. “-”: <50. Red arrow: NobZIP77.

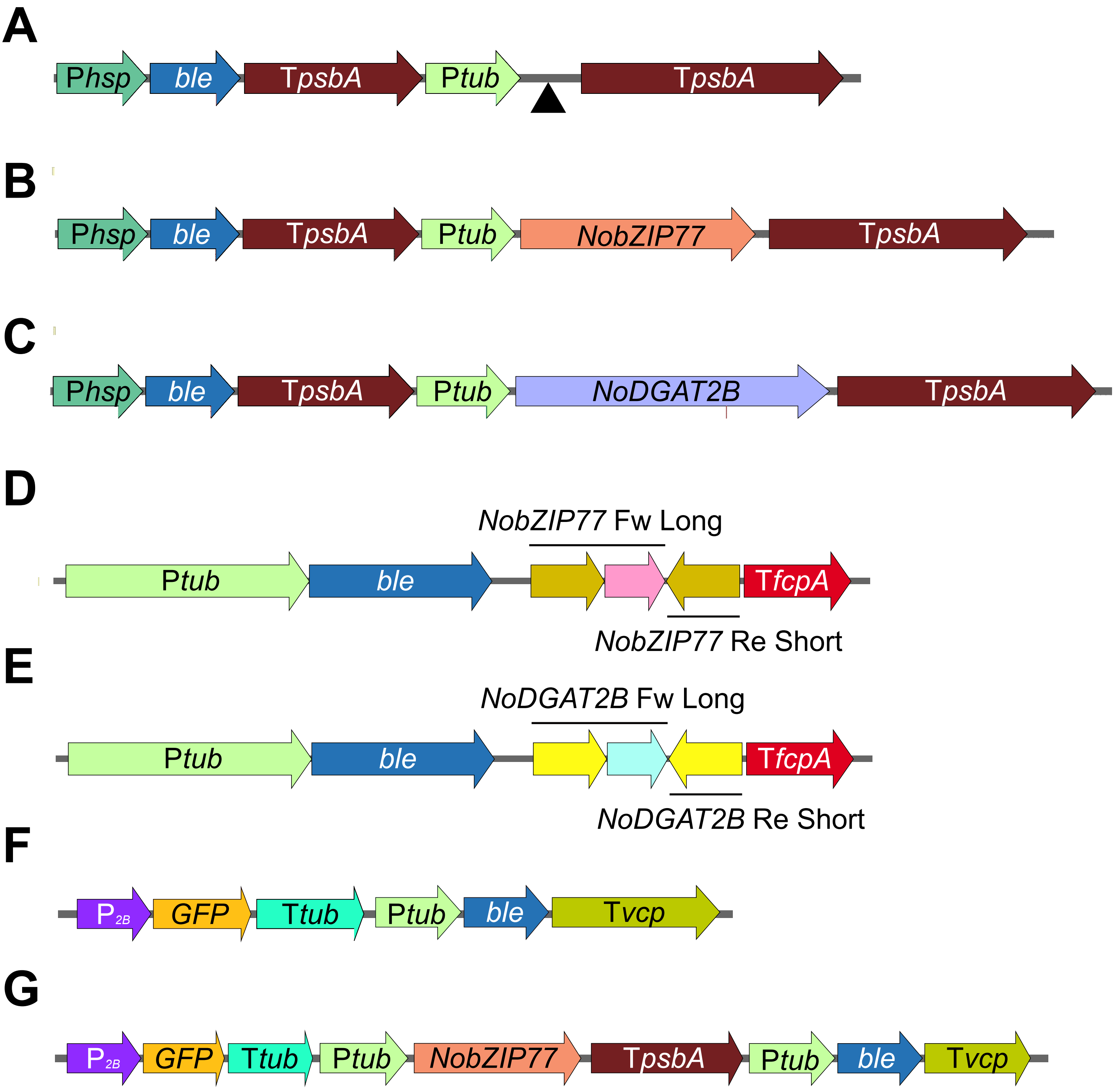

Supplementary Fig. 3. Map of vectors for genetic manipulation of *NobZIP77* and *NoDGAT2B*. The vectors were based on the pXJ450 backbone (**A**). The full-length cDNAs of *NobZIP77* and *NoDGAT2B* were introduced to form pXJ459 (**B**) and pXJ452 (**C**), respectively. (**D-E**) RNAi expression cassette containing inverted repeat of the *NobZIP77* and *NoDGAT2B* for the construction of pXJ448 (**D**) and pXJ432 (**E**). (**F-G**) Vector maps of pXJ545 (**F**) and pXJ546 (**G**). Phsp, promoter of *hsp70A*; Ptub, promoter of *β-tublin*; *ble*, zeocin resistance gene; TpsbA, terminator of *psbA*; TfcpA, terminator of *fcpA*; P_{2B}, promoter of *NoDGAT2B*; Ttub, terminator of *α-tublin*; *GFP*, green fluorescent protein; Tvcp, terminator of *violaxanthin/chlorophyll a binding protein* (VCP). Filled triangle: the multiple cloning sites.

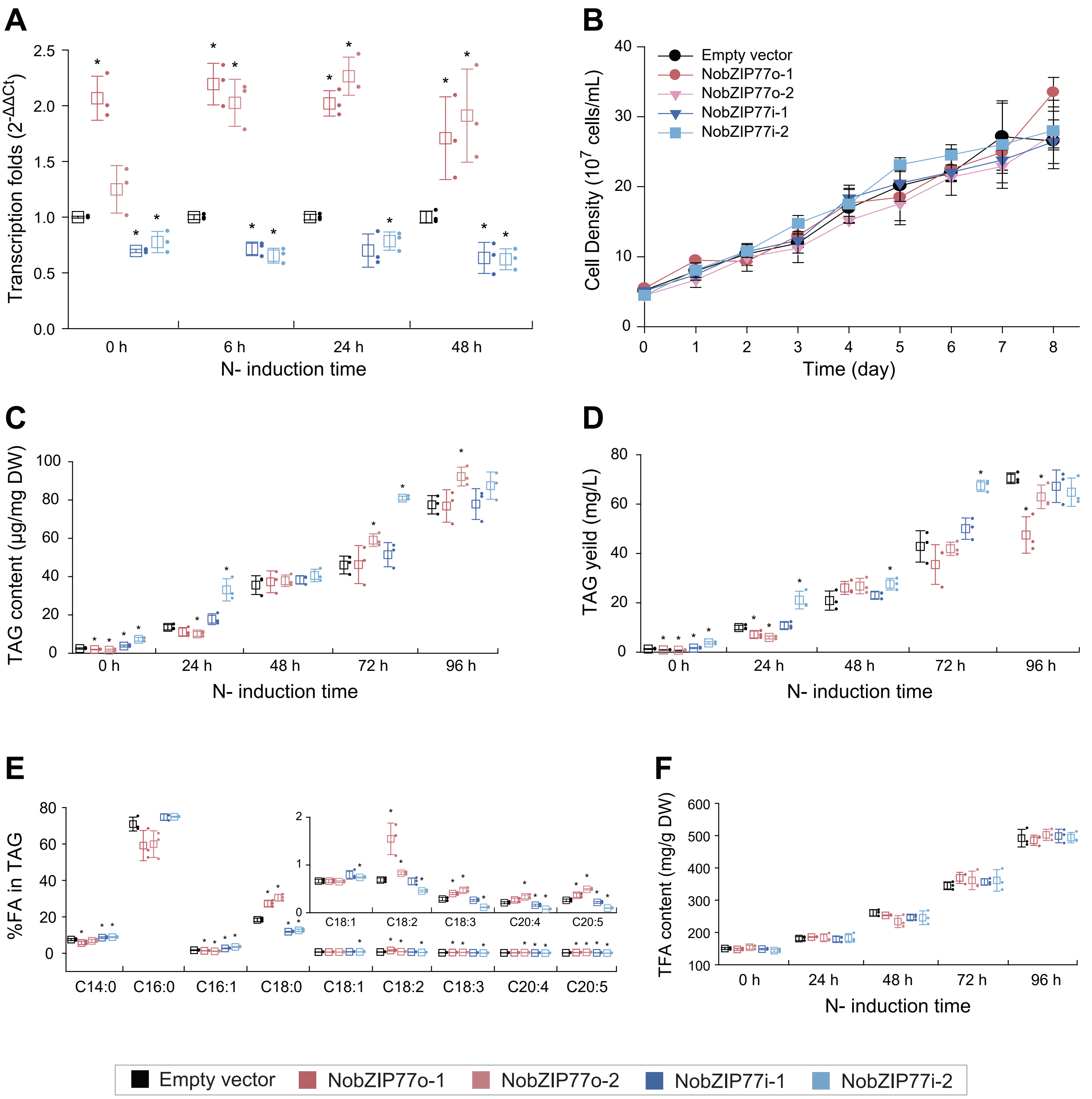

Supplementary Fig. 4. Phenotypes of *NobZIP77* overexpression and knockdown in *Nannochloropsis oceanica*. (**A**) Transcript levels of *NobZIP77* in the two *NobZIP77* overexpression lines (NobZIP77o-1 and NobZIP77o-2) and the two *NobZIP77* knockdown lines (NobZIP77i-1 and NobZIP77i-2) plus an empty vector control (EV) under N+ (0 h) and N- (6 h, 24 h and 48 h after induction), as measured by qRT-PCR. Transcription level of *NobZIP77* was normalized to that of *β-actin*, the internal control. (**B**) Growth kinetics of the *NobZIP77*-transgenic lines and EV. (**C**) TAG content under N+ and N-. (**D**) TAG yield under N+ and N-. (**E**) Fatty-acid composition of TAG under N+. (**F**) The content of total fatty acids (TFAs) under N+ and N-. Data are represented as mean ± SD (n=3 biologically independent samples). *: significant change ($p \leq 0.05$) by one-sided Student's *t*-test versus EV. Source data are provided as a Source Data file.

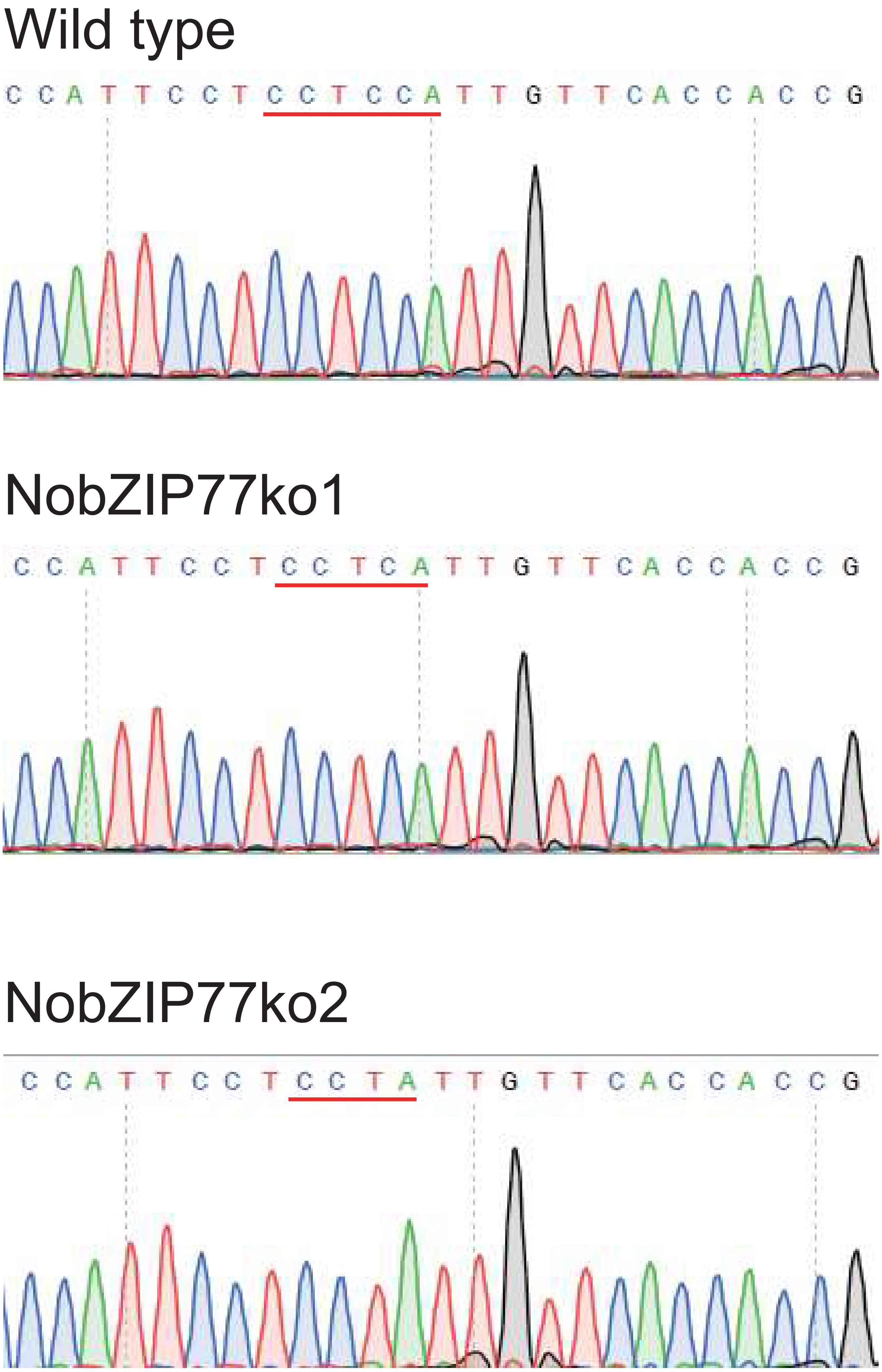

Supplementary Fig. 5. Genome sequences of the editing sites in the *NobZIP77*-knockout lines and wild type of *Nannochloropsis oceanica*. Sequences of the generated *NobZIP77*-CRISPR/Cas9 lines are verified as *NobZIP77*-KO lines with one cytosine (C) deleted in NobZIP77ko1 and two cytosines deleted in NobZIP77ko2.

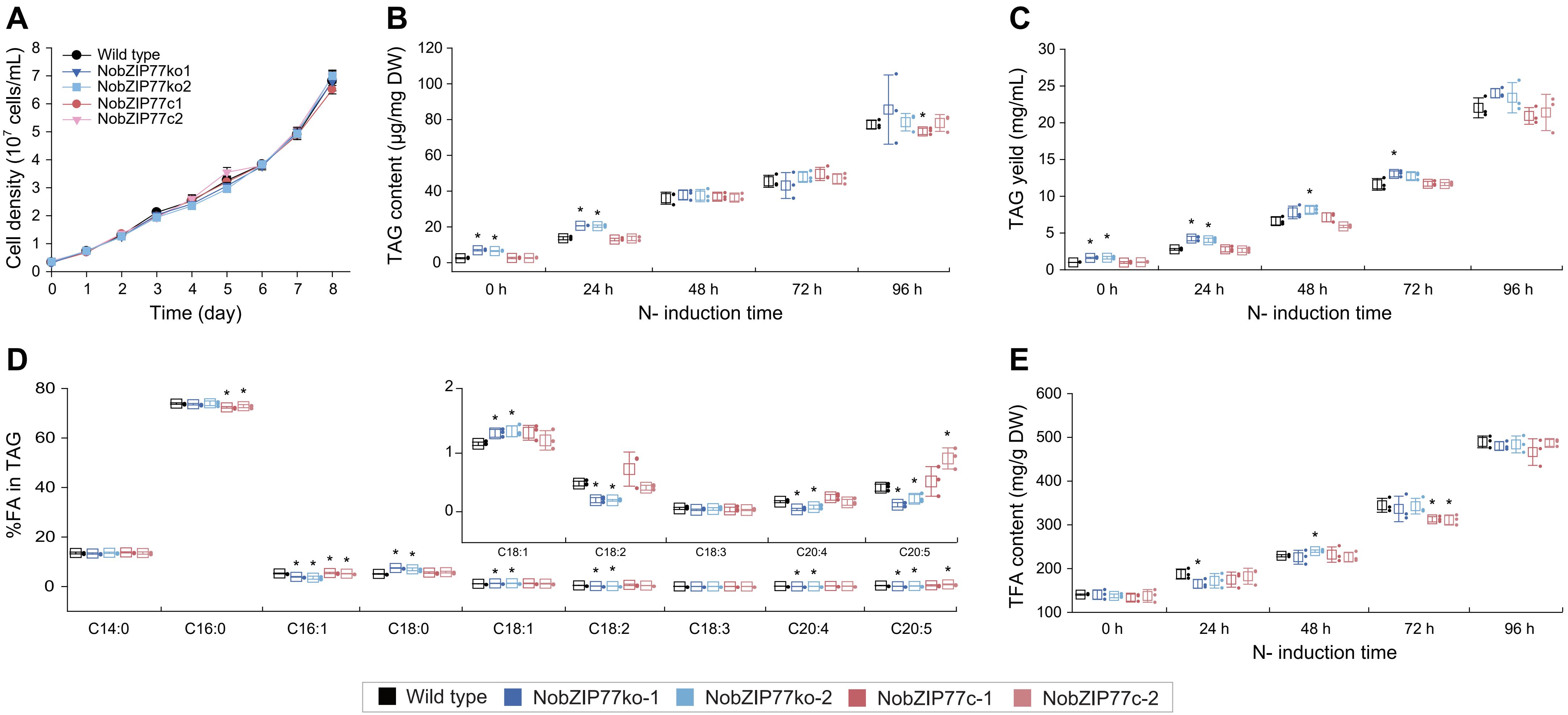

Supplementary Fig. 6. Phenotypes of *NobZIP77* knockout and complementation in *Nannochloropsis oceanica*. (**A**) Growth kinetics of the *NobZIP77* transgenic lines and wild type (WT) under N+. (**B**) TAG content under N+ (0 h) and N- (24 h, 48 h, 72 h and 96 h after induction). (**C**) TAG yield at 0 h, 24 h, 48 h, 72 h and 96 h after onset of N-. (**D**) Fatty-acid composition of TAG at 0 h under N-. (**E**) TFA content at 0 h, 24 h, 48 h, 72 h and 96 h after onset of N-. Data are represented as mean ± SD (n=3 biologically independent samples). *: significant change ($p \leq 0.05$) by one-sided Student's *t*-test versus WT. NobZIP77ko1 and ko2: knockout lines; NobZIP77c1 and c2: complementation lines. Source data are provided as a Source Data file.

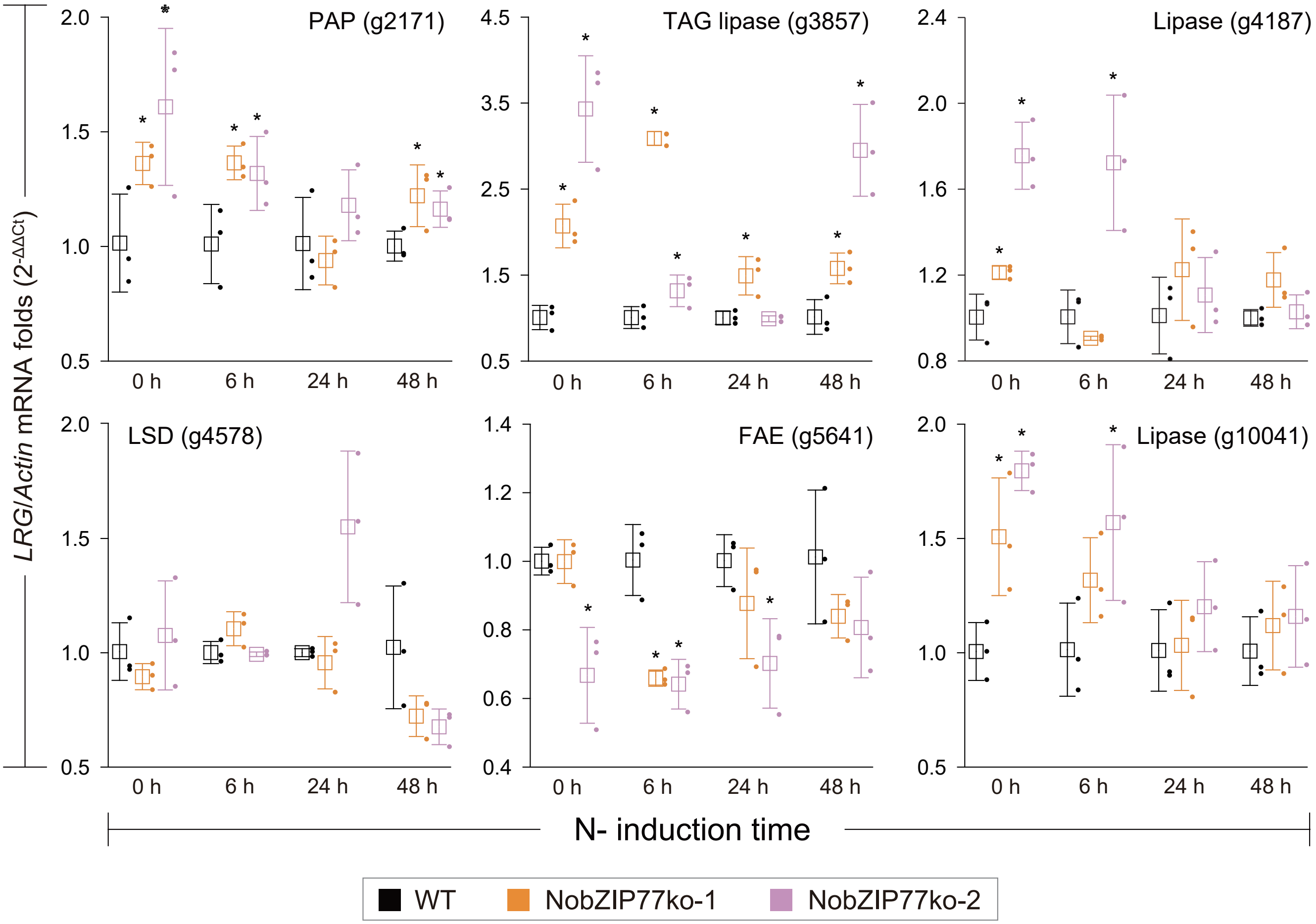

Supplementary Fig. 7. Relative transcript abundance of six lipid-related target genes of NobZIP77 in WT and its knockout lines of NobZIP77ko-1 and NobZIP77ko-2. The cultures were sampled at 0 h, 6 h, 24 h and 48 h under N-. Data are represented as mean ± SD (n=3 biologically independent samples). *: significant change ($p \leq 0.05$) by one-sided Student's *t*-test versus EV. WT, wild type; PAP, Phosphatidic acid phosphohydrolase; LSD, lipid-sensing domain containing protein; FAE, fatty acid elongase. Source data are provided as a Source Data file.

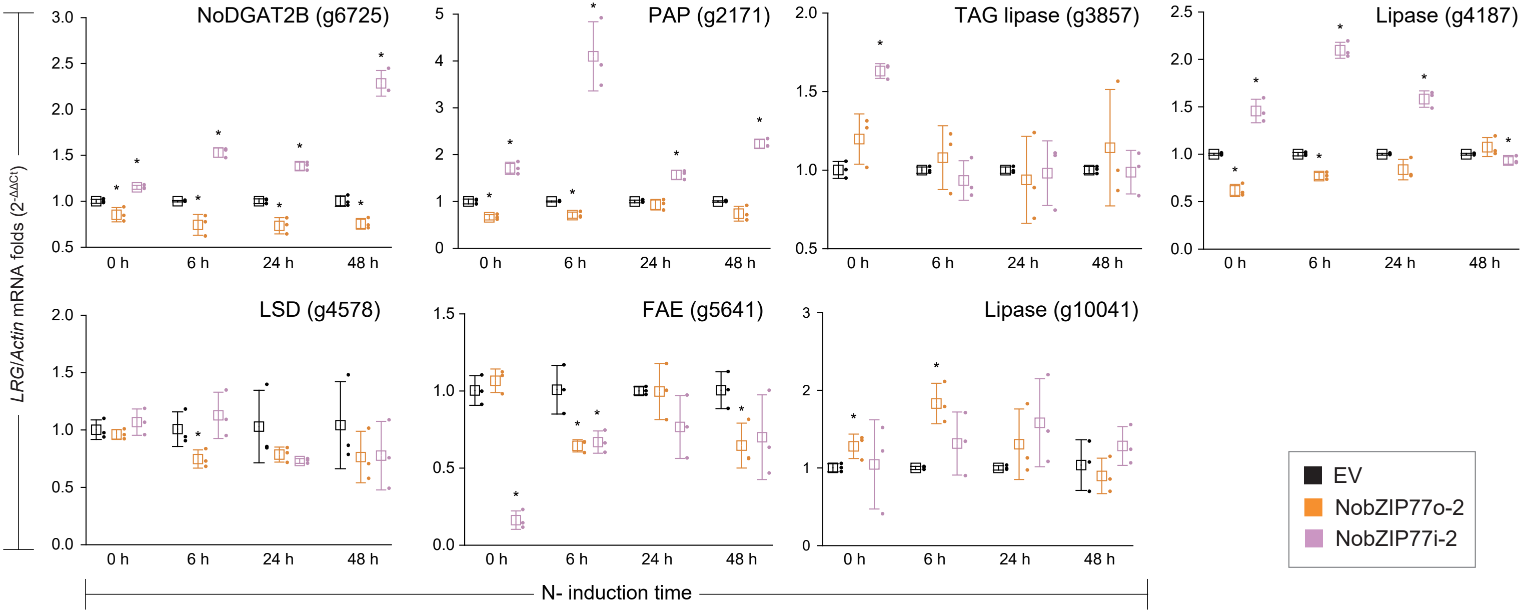

Supplementary Fig. 8. Relative transcript abundance of seven lipid-related target genes of NobZIP77 in EV and its knockdown and overexpression lines of NobZIP77o-2 and NobZIP77i-2. The cultures were sampled at 0 h, 6 h, 24 h and 48 h under N-. Data are represented as mean ± SD (n=3 biologically independent samples). *: significant change ($p \leq 0.05$) by one-sided Student's *t*-test versus EV. EV, empty vector control; PAP, Phosphatidic acid phosphohydrolase; LSD, lipid-sensing domain containing protein; FAE, fatty acid elongase. Source data are provided as a Source Data file.

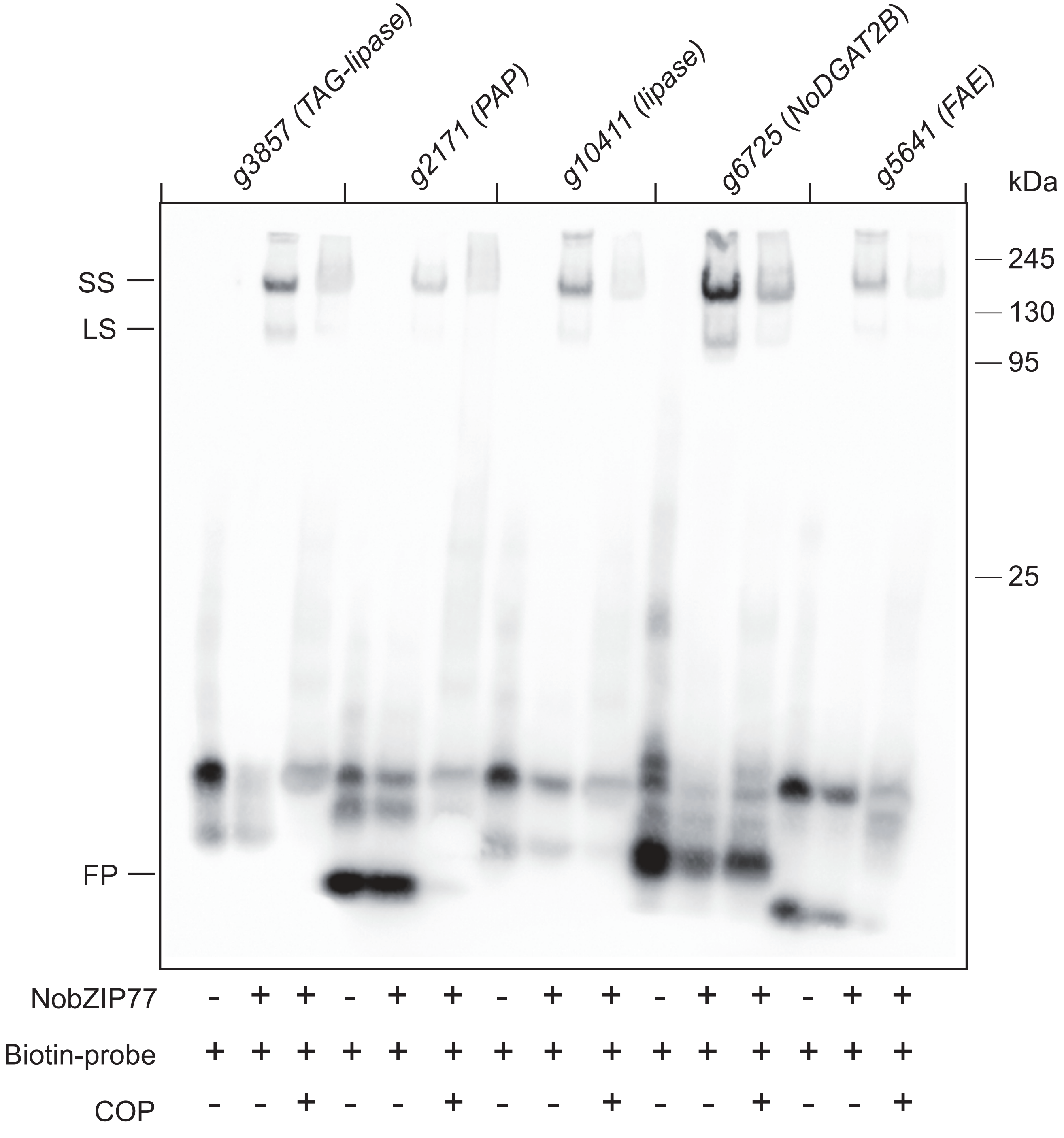

Supplementary Fig. 9. EMSA-based validation of NobZIP77-target genes. EMSA results revealed that NobZIP77 directly binds to the promoters of *NoDGAT2B*, *g5641* (*FAE*), *g10411* (*lipase*), *g2171* (*PAP*) and *g3857* (*TAG-lipase*). Unlabelled DNA of the promoters in 100-fold molar excess was treated with the NobZIP77 protein. PAP, Phosphatidic acid phosphohydrolase; FAE, fatty acid elongase; LS, lower shift complex (a 1:1 complex of DNA and the bZIP dimer); SS, super shift complex; FP, free-DNA probe; COP, competitor oligonucleotide primer. The experiments were repeated three times. Source data are provided as a Source Data file.

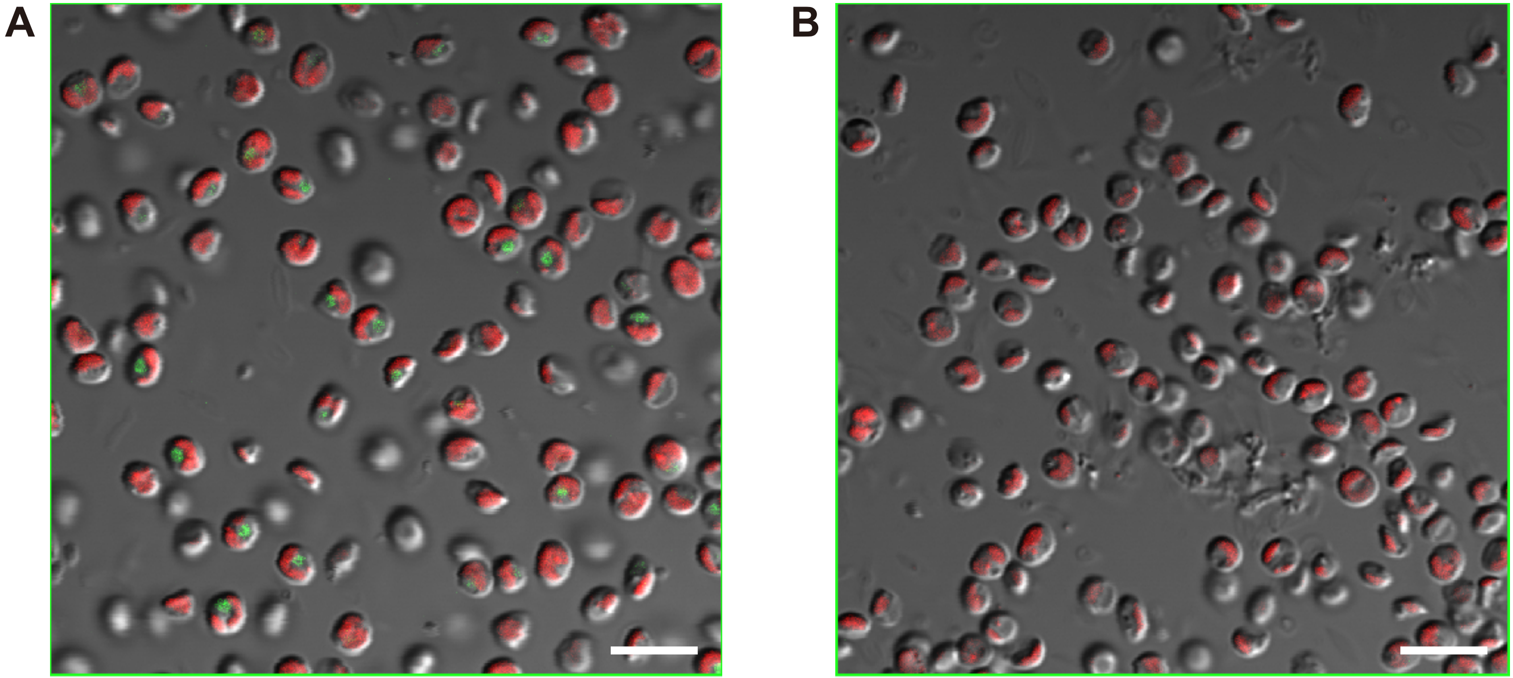

Supplementary Fig. 10. Confocal GFP-fluorescence images of GFP-*NobZIP77*-transformed *Nannochloropsis oceanica* (OE1; A) and WT (B). Green, GFP-fluorescence; red, plastid autofluorescence ; scale bar, 5 μm. The experiments were repeated three times.

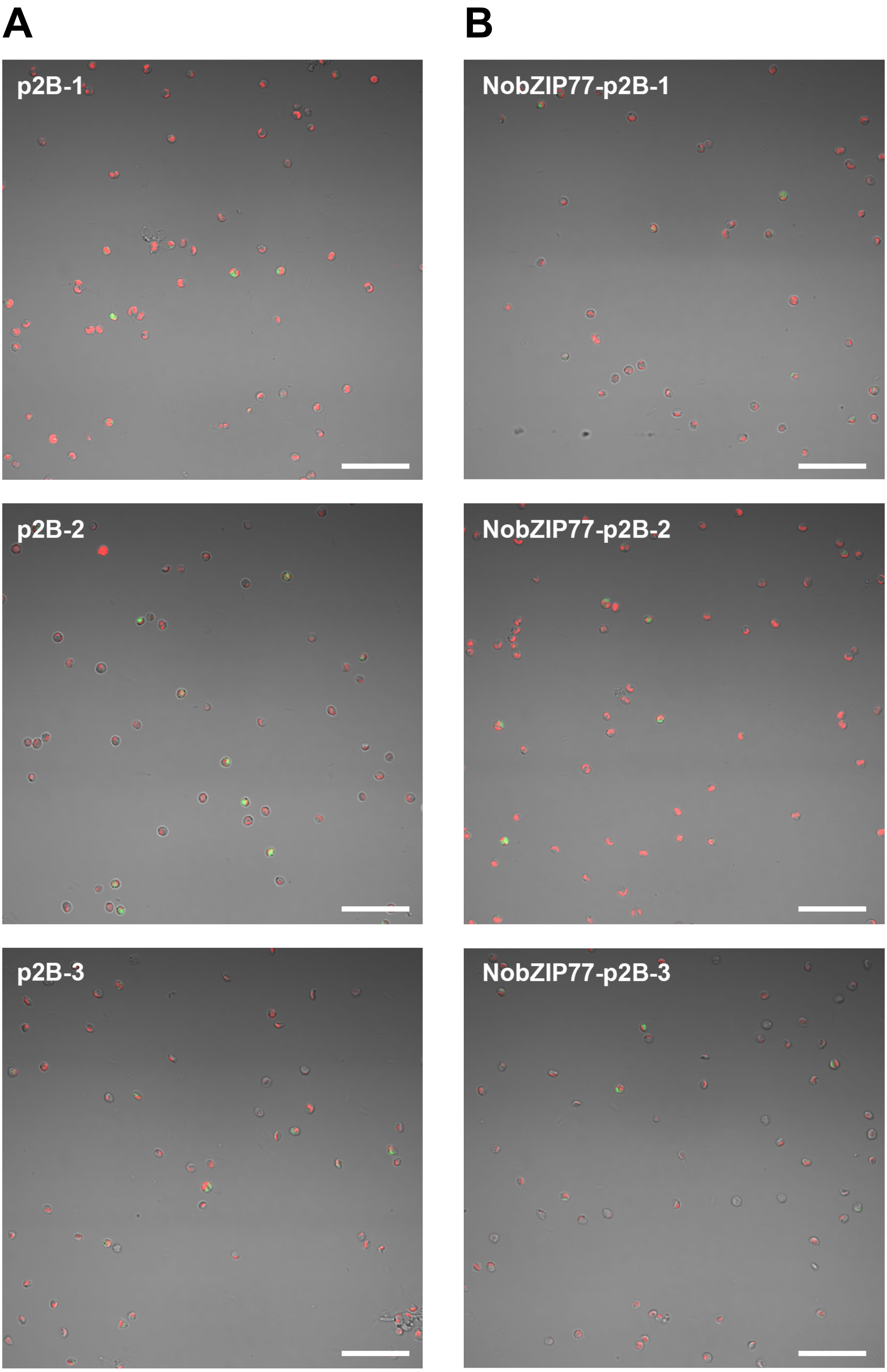

Supplementary Fig. 11. Confocal GFP-fluorescence images of *NoDGAT2B*-promoter-transformed (A) and NobZIP77-*NoDGAT2B*-promoter-transformed *Nannochloropsis oceanica* (B). Three lines of each transformed cassette were presented, which were also used for the quantification of GFP-fluorescence intensity in **Fig. 2C**. Green, GFP-fluorescence; red, plastid autofluorescence ; scale bar, 10 μm. The experiments were repeated three times.

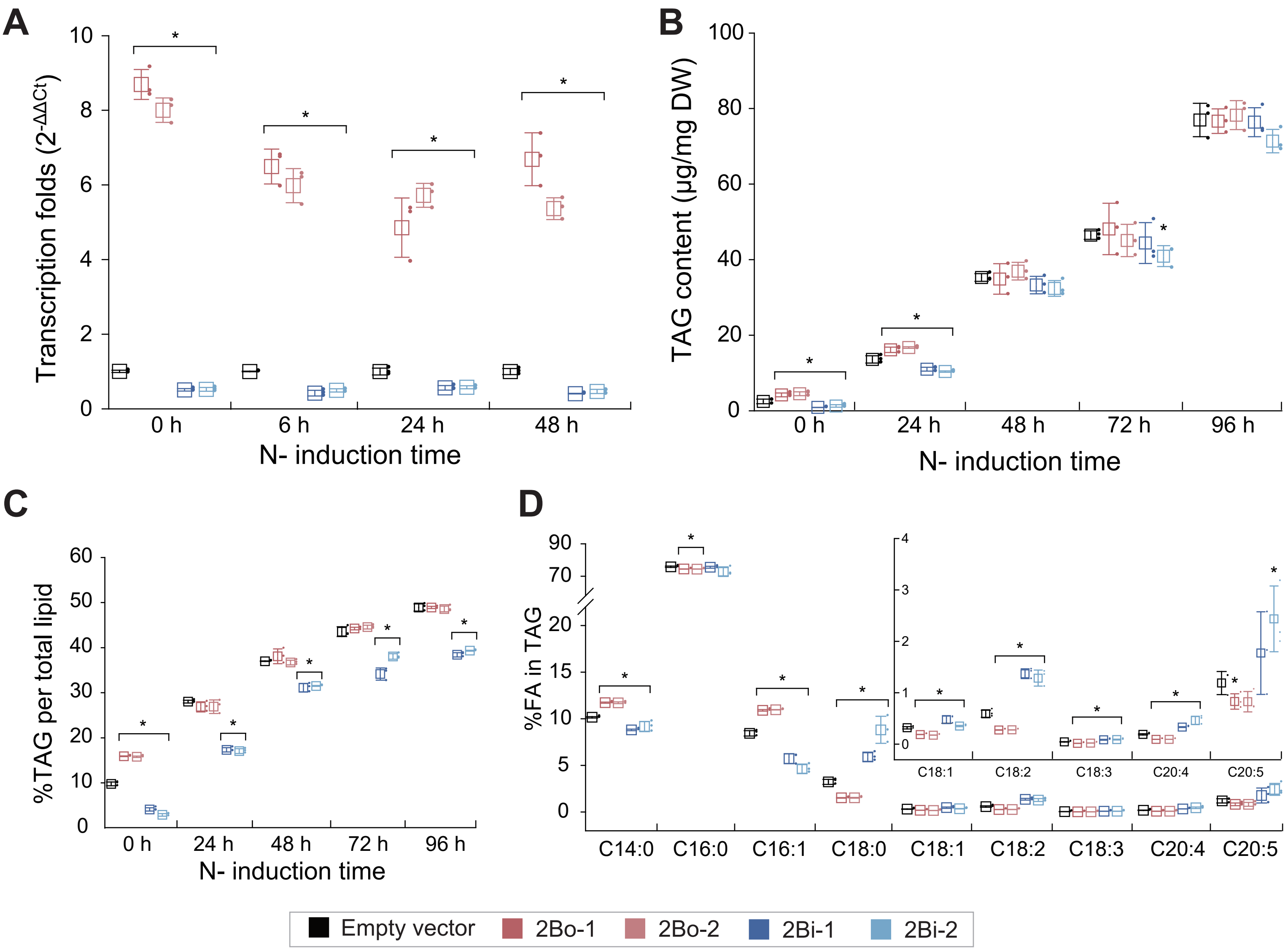

Supplementary Fig. 12. Phenotypes of *NoDGAT2B* overexpression and knockdown in *Nannochloropsis oceanica*. (**A**) Transcript levels of *NoDGAT2B* in the two *NoDGAT2B* overexpression lines (2Bo-1 and 2Bo-2) and the two *NoDGAT2B* knockdown lines (2Bi-1 and 2Bi-2) plus an empty vector control (EV) under N+ (0 h) and N- (6 h, 24 h and 48 h after induction), as measured by qRT-PCR. Transcription level of *NoDGAT2B* was normalized by that of *β-actin*, the internal control. (**B**) TAG content (µg/mg DW) under N+ (0 h) and N- (24 h, 48 h, 72 h and 96 h after induction, considering a ~24 h lag between change in abundance of TAG-synthesis-related transcripts and TAG content in *N. oceanica*). (**C**) TAG content (%TAG per total lipid) under N+ and N-. (**D**) Fatty-acid composition of TAG under N+. Data are represented as mean ± SD (n=3 biologically independent samples). *: significant change ($p \leq 0.05$) by one-sided Student's *t*-test versus EV. Source data are provided as a Source Data file.

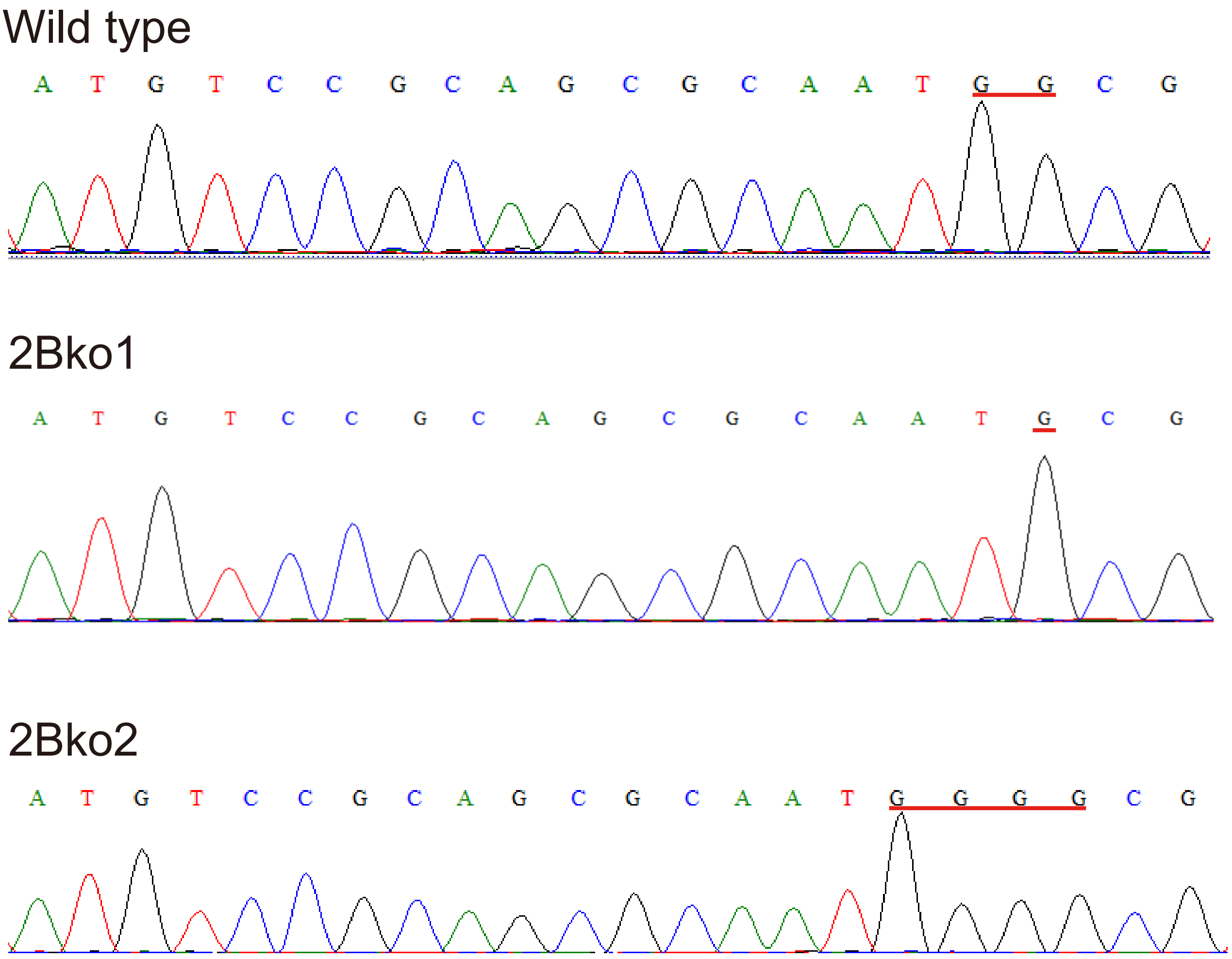

Supplementary Fig. 13. Genome sequences of the editing sites in the *NoDGAT2B* knockout lines and wild type. Sequences of the generated *NoDGAT2B*-CRISPR/Cas9 lines confirmed the mutants as KO lines with one G deleted in 2Bko1 and two G inserted in 2Bko2.

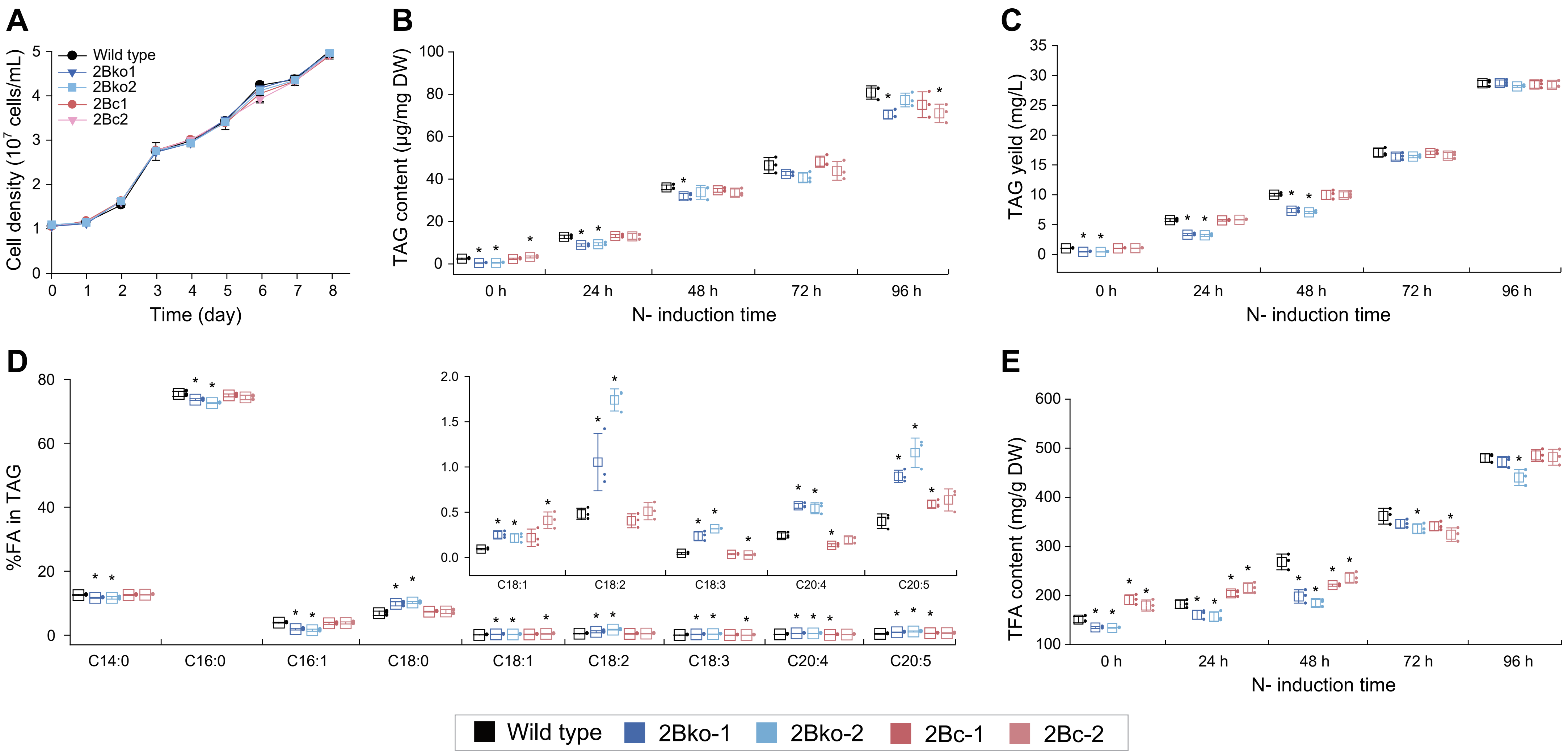

Supplementary Fig. 14. Phenotypes of *NoDGAT2B* knockout and complementation in *Nannochloropsis oceanica*. (**A**) Growth kinetics of the *NoDGAT2B* transgenic lines and WT under N+. (**B**) TAG content under N+ (0 h) and N- (24 h, 48 h, 72 h and 96 h after induction). (**C**) TAG yield at 0 h, 24 h, 48 h, 72 h and 96 h after onset of N-. (**D**) Fatty-acid composition of TAG at 0 h under N-. (**E**) TFA content at 0 h, 24 h, 48 h, 72 h and 96 h after onset of N-. Data are represented as mean ± SD (n=3 biologically independent samples). *: significant change ($p \leq 0.05$) by one-sided Student's *t*-test versus WT. 2Bko1 and 2Bko2: knockout lines; 2Bc1 and c2: complementation lines. Source data are provided as a Source Data file.

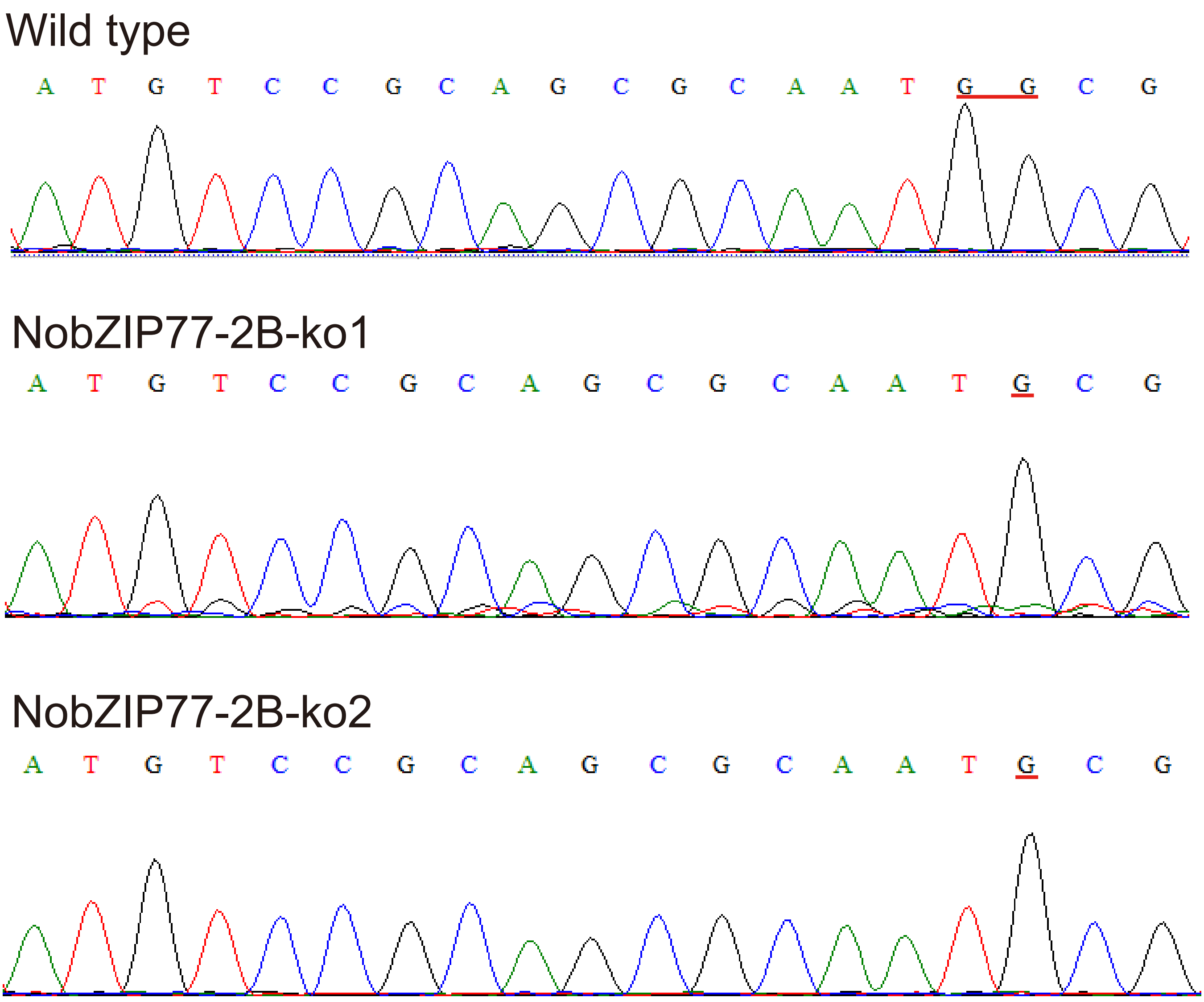

Supplementary Fig. 15. Genome sequences of the editing sites in the *NobZIP77-NoDGAT2B* double-knockout lines and the wild type. These results confirmed the expected double-knockout events (one G deleted from the *NoDGAT2B*-edited sites in the NobZIP77-2B-ko1 and NobZIP77-2B-ko2 lines; DNA sequences of the *NobZIP77*-edited sites are identical to those of NobZIP77ko-1 as shown in **Supplementary Fig. 5**).

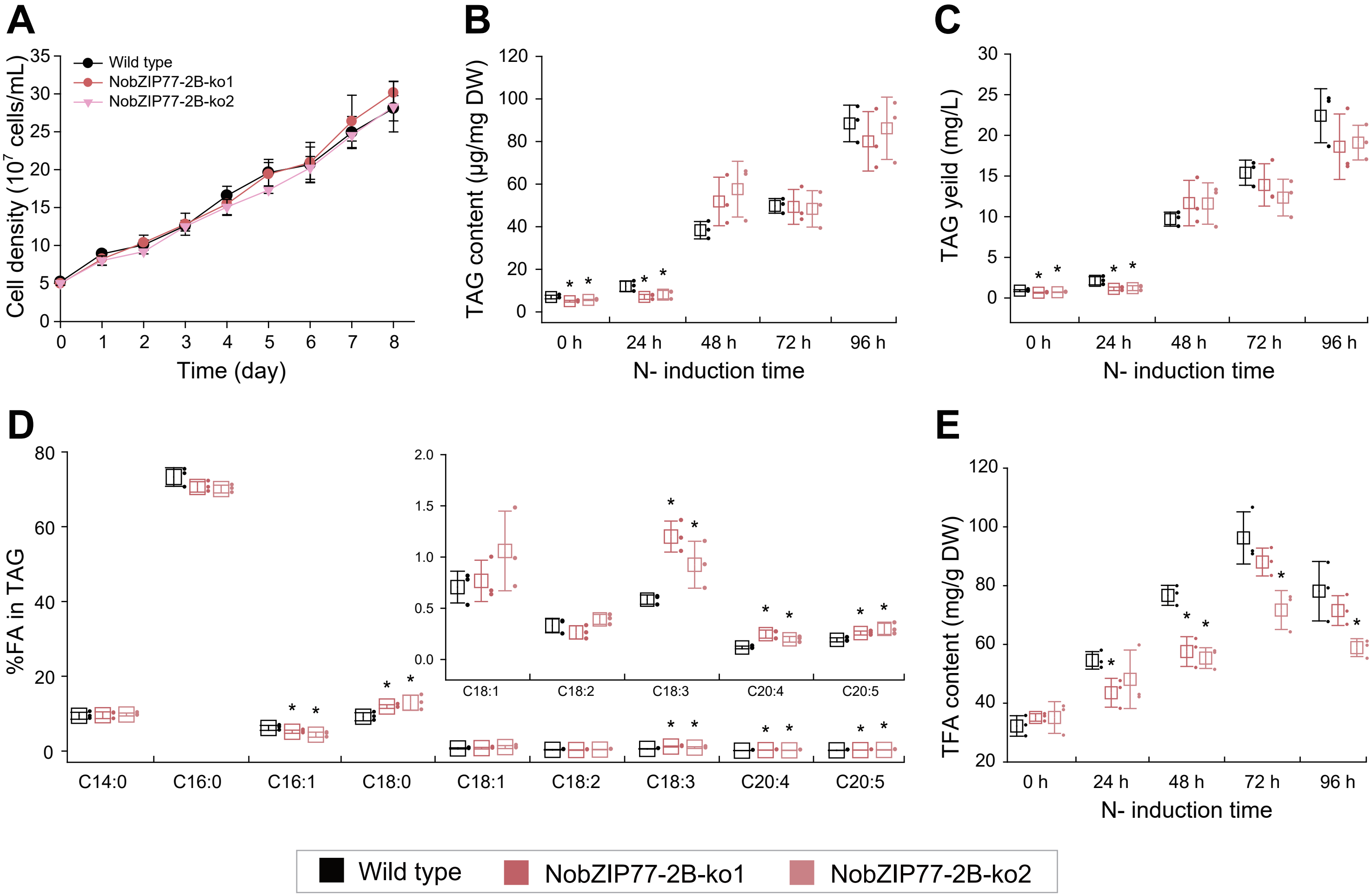

Supplementary Fig. 16. Phenotypes of *NobZIP77-NoDGAT2B* double-knockout in *Nannochloropsis oceanica*. (**A**) Growth kinetics of the double-knockout lines and WT under N+. (**B**) TAG content under N+ (0 h) and N- (24 h, 48 h, 72 h and 96 h after induction). (**C**) TAG yield at 0 h, 24 h, 48 h, 72 h and 96 h after onset of N-. (**D**) Fatty-acid composition of TAG at 0 h under N-. (**E**) TFA content at 0 h, 24 h, 48 h, 72 h and 96 h after onset of N-. Data are represented as mean ± SD (n=3 biologically independent samples). *: significant change ($p \leq 0.05$) by one-sided Student's *t*-test versus WT. NobZIP77-2B-ko1 and NobZIP77-2B-ko2: the two double-knockout lines. Source data are provided as a Source Data file.

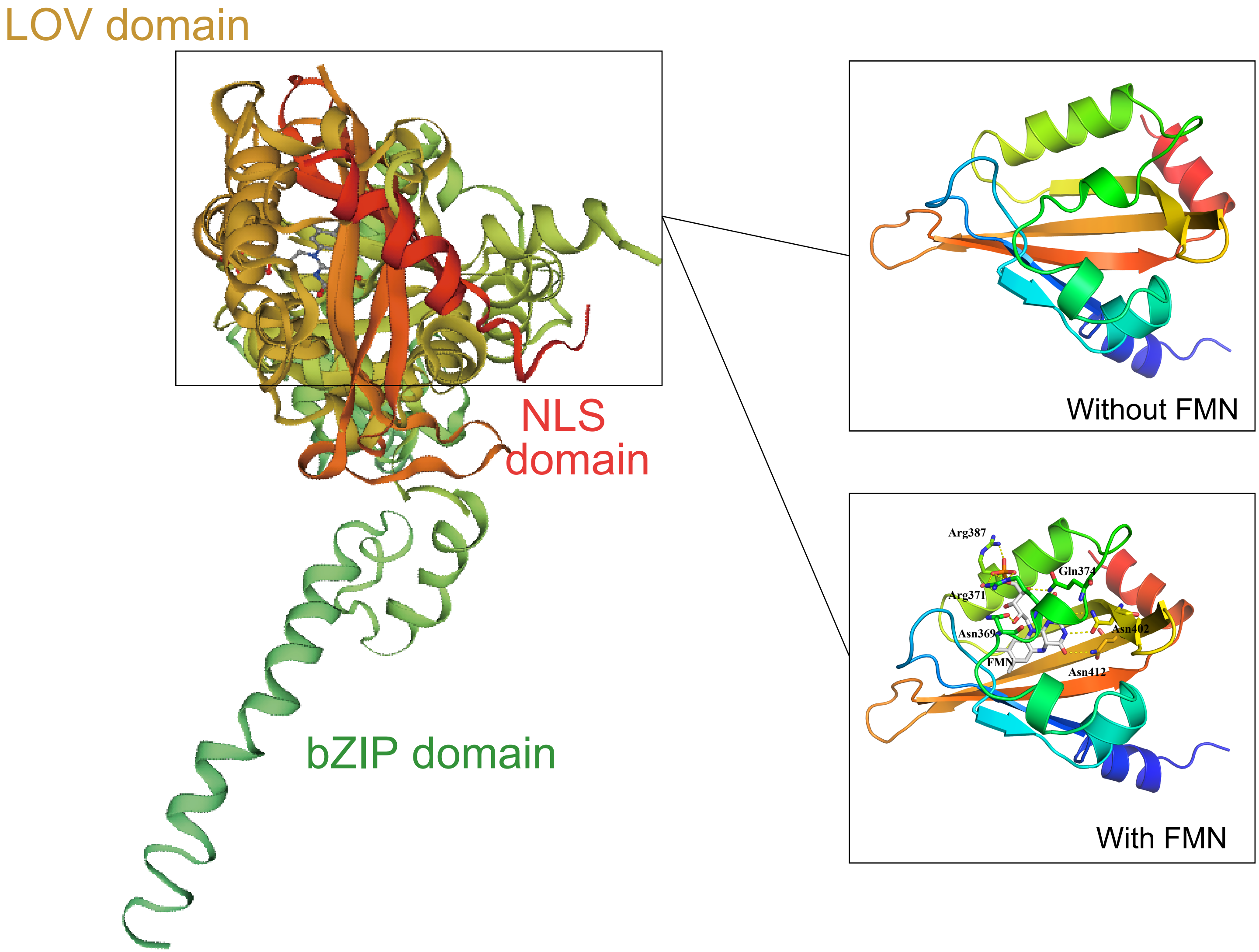

Supplementary Fig. 17. Structural models of NobZIP77 with or without (FMN). FMN: flavin mononucleotide. The tertiary structure of NobZIP77 (249-391) was modeled using a Circadian locomotor output cycles protein kaput as initial template (15.44% sequence identity).

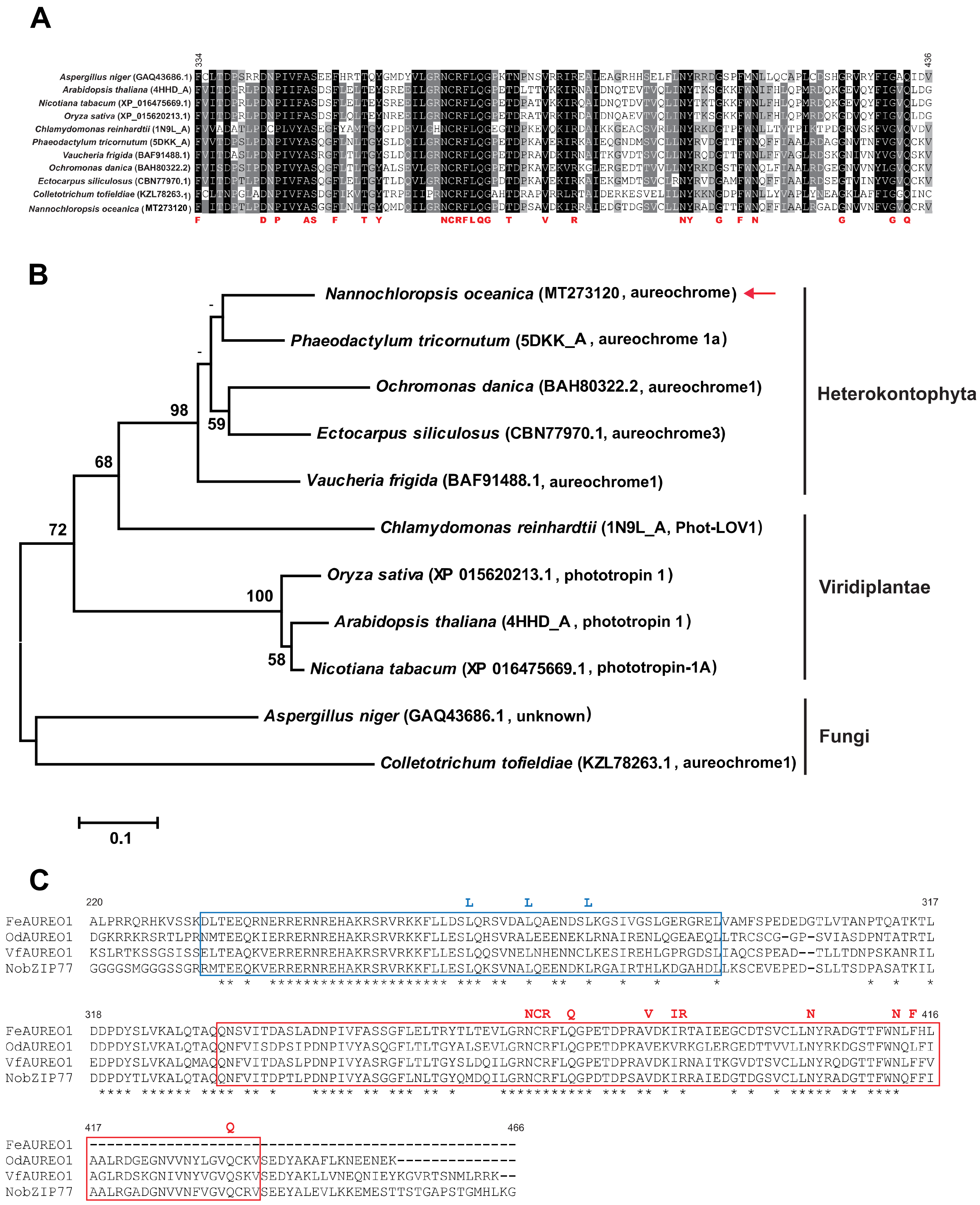

Supplementary Fig. 18. Phylogenetic analysis of the LOV domain of NobZIP77. (**A**) Alignment of amino acid sequences among LOV domains from higher plants, fungi and microalgae. Accession numbers are shown. Bold residues below the sequences: the conserved sites. Numbers above the sequences: amino acid position on NobZIP77. (**B**) Cladogram of selected LOV protein sequences from higher plants, fungi and microalgae. Neighbor-joining (NJ) method was used for tree construction. Cladogram was plotted based on actual branch length. GenBank accession numbers are provided in brackets. The numbers beside the branch stand for bootstrap value for NJ. “-” indicated values < 50. Red arrow represents NobZIP77. (**C**) Aligned sequences of the AUREO1s from *N. oceanica* and its orthologs. Blue and red frames indicate bZIP and LOV domains, respectively. Heptad leucine residues of bZIP domains are in blue, while the eleven conserved amino acid residues that are associated with flavin binding are in red. The asterisks indicate identical amino acids among the following AUREO1s: FeAUREO1 (*Fucus evanescens*, BAH80320.1), OdAUREO1 (*Ochromonas danica*, BAH80322.2), VfAUREO1 (*Vaucheria frigida*, BAF91488.1) and NobZIP77 (*N. oceanica*, MT273120).

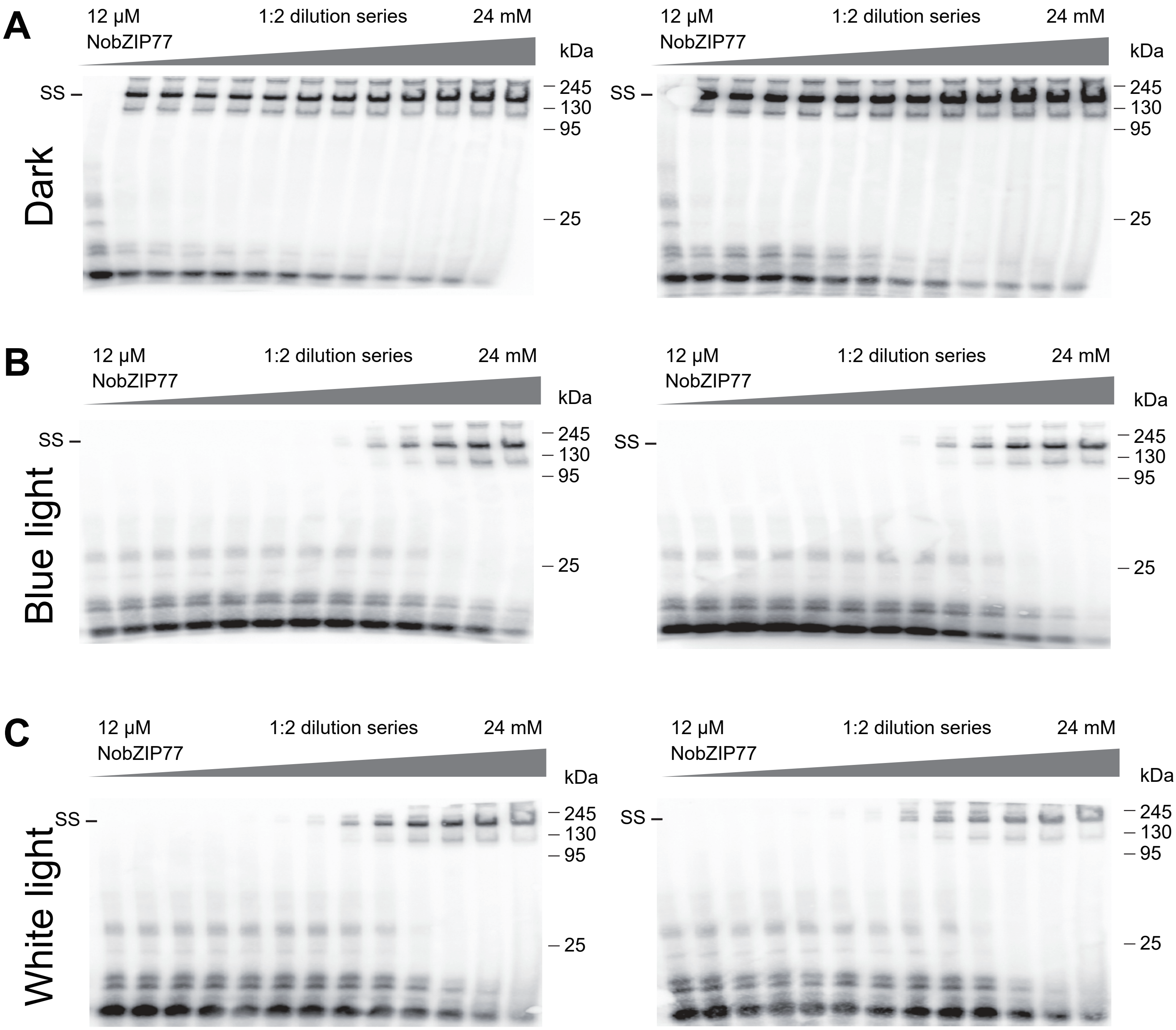

Supplementary Fig. 19. Blue light reduces the binding of NobZIP77 to the *NoDGAT2B* promoter. EMSAs under dark (**A**), blue light (BL; **B**) or white light (WL; **C**) in the presence of 5 nM *NoDGAT2B* promoter DNA and varying amounts of purified NobZIP77 (a 1:2 dilution series of NobZIP77 with the maximum concentration of 24mM). SS (super shift complex) was used for optical density quantification, and compared among the three conditions of darkness, BL and WL. The experiments were repeated two times. Source data are provided as a Source Data file.

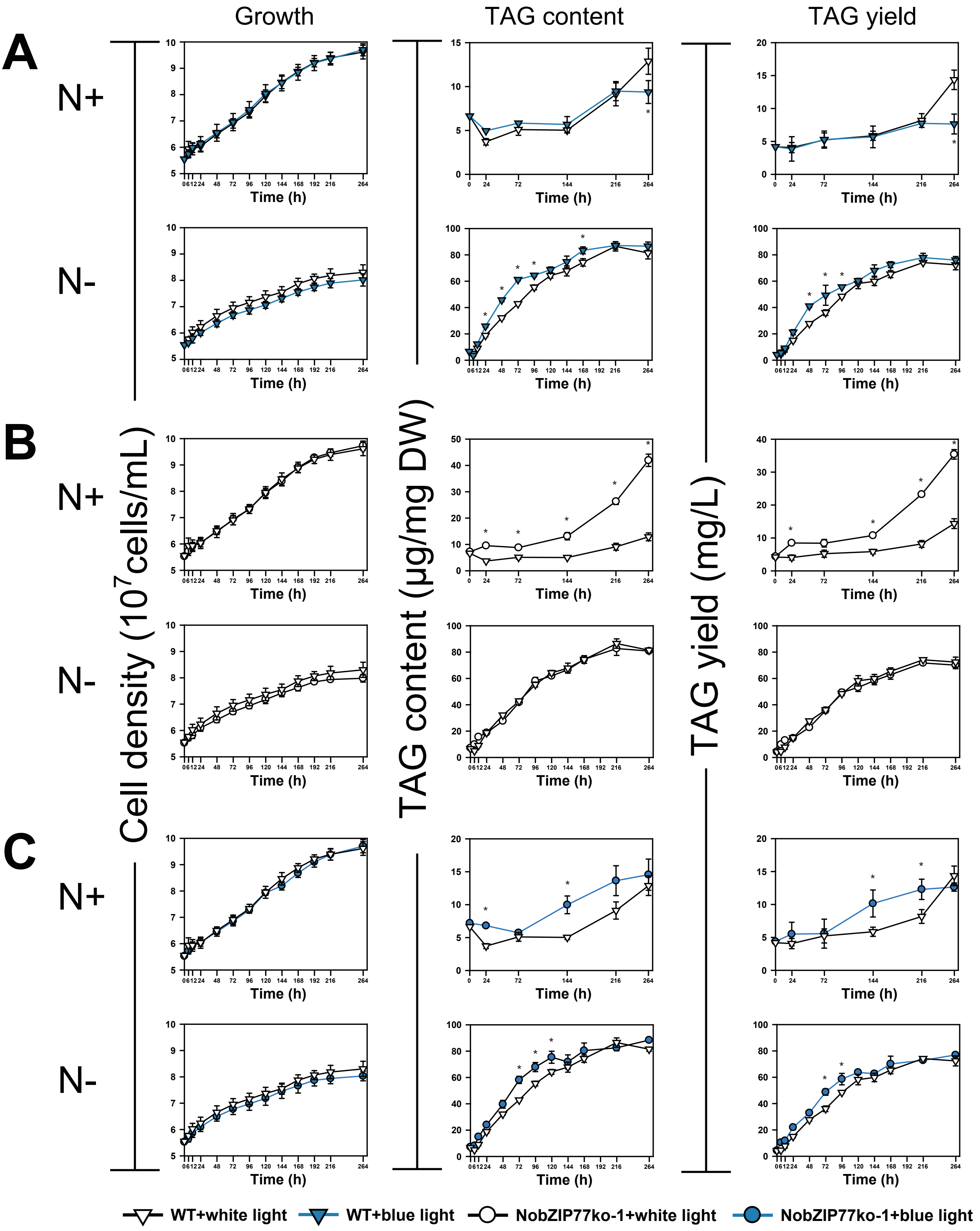

Supplementary Fig. 20. Phenotypes of *Nannochloropsis oceanica* NobZIP77 mutant and WT cultivated under blue light. Three independent cultures were sampled for a period of 11 days. For each of the three independent sets of samples, TAG contents and yields were quantified by single-cell Raman microscpectroscopy, based the protocols that we have published. The results yield very small error bars. Growth kinetics (cell density, 10^7 cells/mL), TAG content (μg/mg DW) and TAG yield (mg/L) were compared for (**A**) between wild type (WT) under white light (WL) and WT under blue light (BL), (**B**) WT and NobZIP77ko-1 under WL, and (**C**) WT under WL and NobZIP77ko-1 under BL, under both nitrogen-replete (N+) and nitrogen-depleted (N-) conditions. Data are represented as mean ± SD (n=3 biologically independent samples). *: significant change ($p \leq 0.05$) by one-sided Student's *t*-test. Source data are provided as a Source Data file.

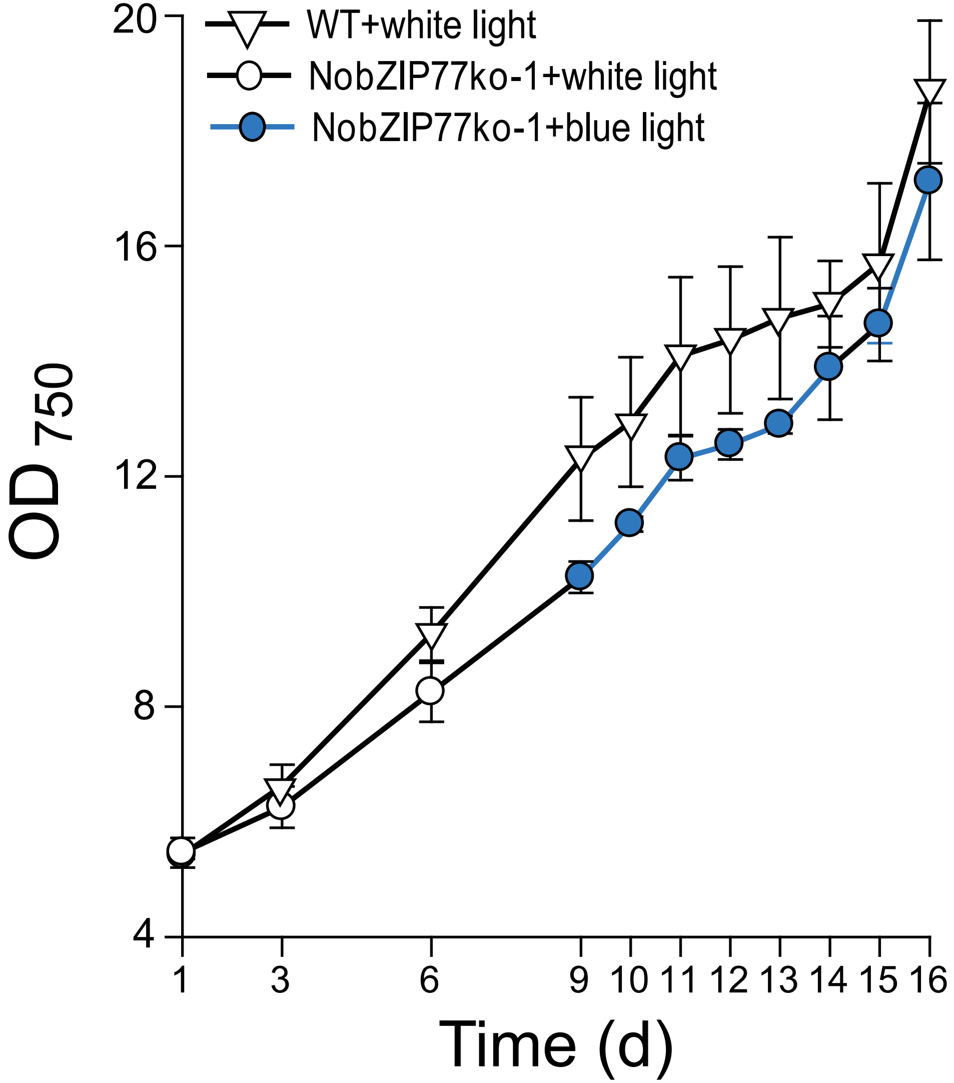

Supplementary Fig. 21. Comparing *N. oceanica* growth kinetics (OD_{750}) between the blue-light induced oil production (BLIO) strategy and the control strategy. BLIO: *NobZIP77*-knockout line cultured for nine days under white light and then seven days under blue light. Control: wild-type (WT) *N. oceanica* under white light (WL) over 16 days of cultivation. Data are represented as mean ± SD (n=3 biologically independent samples). Source data are provided as a Source Data file.

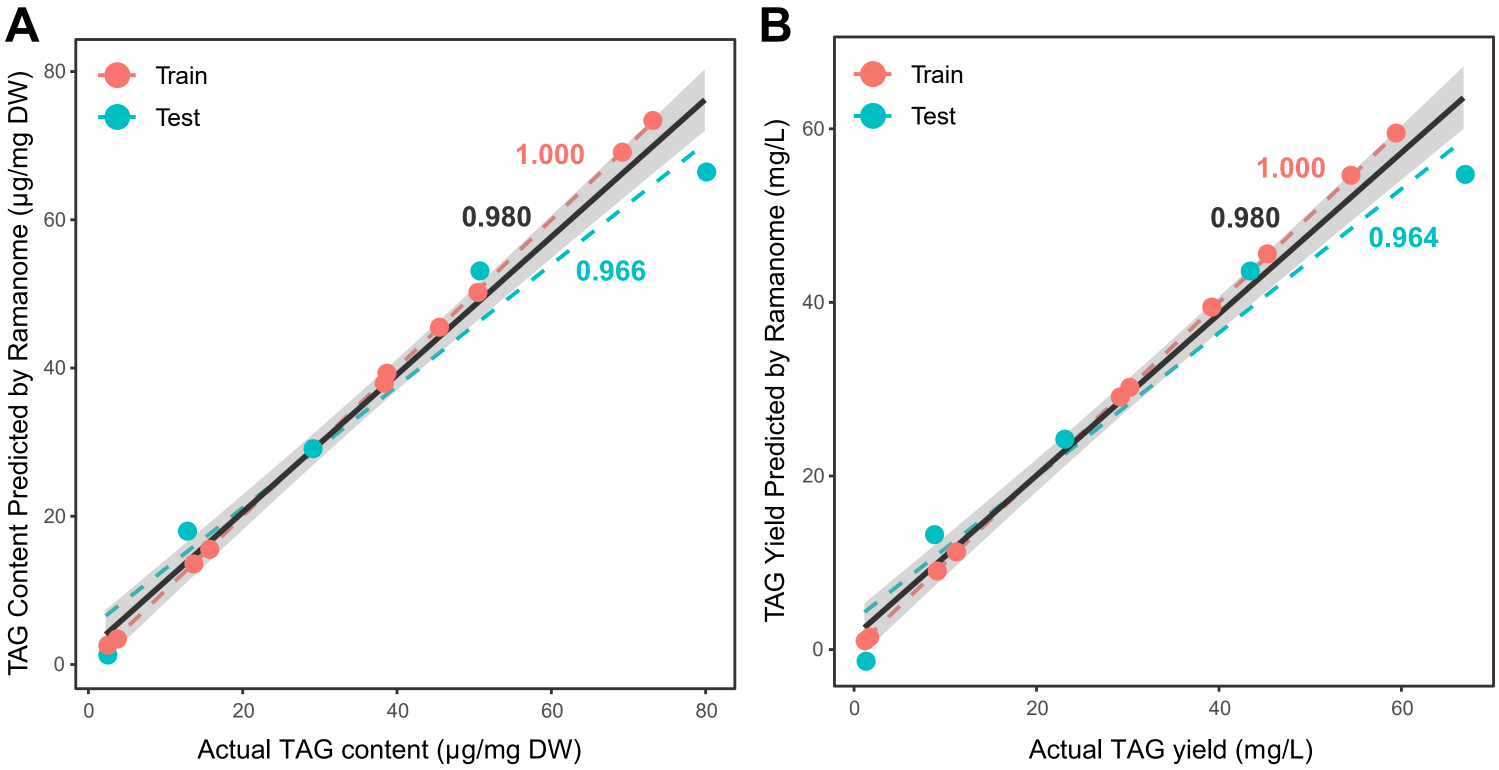

Supplementary Fig. 22. Quantification of TAG content (A) and yield (B) via ramanome. The TAG content (µg/mg DW) and yield (mg/L) predicted using PLSR models via ramanome (Y axis; two of the three ramanomes were used for train and one for test) were plotted versus the corresponding value measured with TLC-GC-MS at the population level (X axis). PLSR: partial least square regression. TLC-GC-MS: Thin-layer chromatography coupled with gas chromatography-mass spectrometry. Correlation coefficients (R^2) were shown for train data (red), test data (green) and all data (black). Source data are provided as a Source Data file.

References

1. You, W.X. et al. Integration of proteome and transcriptome refines key molecular processes underlying oil production in *Nannochloropsis oceanica*. Biotechnol. Biofuels 13, 109 (2020).
2. Li, J. et al. Choreography of transcriptomes and lipidomes of Nannochloropsis reveals the mechanisms of oil synthesis in microalgae. *Plant Cell* 26, 1645-1665 (2014).
