# Supplementary material for: Exploring a blue-light-sensing transcription factor to double the peak productivity of oil in Nannochloropsis oceanica
Source: Nat Commun. 2022 Mar 29;13:1664. doi: 10.1038/s41467-022-29337-x (PMC8964759; doi:10.1038/s41467-022-29337-x)
Supplement: Supplementary file 3 — Description of Additional Supplementary Files [file 41467_2022_29337_MOESM3_ESM.pdf]

File Name: Supplementary Data 1
Description: N-depletion induced TFs and their putative LRGs in *N. oceanica*.

File Name: Supplementary Data 2
Description: Primers used in the genetic manipulation of *NobZIP77* and *NoDGAT2B*.

File Name: Supplementary Data 3
Description: Primers used in the EMSA assays.
