# Supplementary material for: Exploring a blue-light-sensing transcription factor to double the peak productivity of oil in Nannochloropsis oceanica
Source: Nat Commun. 2022 Mar 29;13:1664. doi: 10.1038/s41467-022-29337-x (PMC8964759; doi:10.1038/s41467-022-29337-x)
Supplement: Supplementary file 7 — Reporting Summary [file 41467_2022_29337_MOESM7_ESM.pdf]

nature portfolio

Corresponding author(s): Jian XU

Last updated by author(s): Mar 11, 2022

Reporting Summary

Nature Portfolio wishes to improve the reproducibility of the work that we publish. This form provides structure for consistency and transparency in reporting. For further information on Nature Portfolio policies, see our Editorial Policies and the Editorial Policy Checklist.

Statistics

For all statistical analyses, confirm that the following items are present in the figure legend, table legend, main text, or Methods section.

n/a	Confirmed	
☐	☒	The exact sample size (*n*) for each experimental group/condition, given as a discrete number and unit of measurement
☐	☒	A statement on whether measurements were taken from distinct samples or whether the same sample was measured repeatedly
☐	☒	The statistical test(s) used AND whether they are one- or two-sided<br>*Only common tests should be described solely by name; describe more complex techniques in the Methods section.*
☐	☒	A description of all covariates tested
☐	☒	A description of any assumptions or corrections, such as tests of normality and adjustment for multiple comparisons
☐	☒	A full description of the statistical parameters including central tendency (e.g. means) or other basic estimates (e.g. regression coefficient) AND variation (e.g. standard deviation) or associated estimates of uncertainty (e.g. confidence intervals)
☐	☒	For null hypothesis testing, the test statistic (e.g. *F*, *t*, *r*) with confidence intervals, effect sizes, degrees of freedom and *P* value noted<br>*Give P values as exact values whenever suitable.*
☐	☒	For Bayesian analysis, information on the choice of priors and Markov chain Monte Carlo settings
☐	☒	For hierarchical and complex designs, identification of the appropriate level for tests and full reporting of outcomes
☐	☒	Estimates of effect sizes (e.g. Cohen's *d*, Pearson's *r*), indicating how they were calculated

Our web collection on statistics for biologists contains articles on many of the points above.

Software and code

Policy information about availability of computer code

Data collection	FV10-ASW 2.0 Viewer (Olympus), Fusion solo 6s (Vilber), MSD chemstation E.02.02.1431 (Agilent Technology), Labspec 5 (Horiba)
Data analysis	FV10-ASW 2.0 Viewer (Olympus), Image J (NIH), MSD chemstation E.02.02.1431 (Agilent Technology), Sigmaplot 12.0, MEME version 5.3.3, MUSCLE version 3.8.31, BioEdit version 7.0.5.3, MEGA4.1, PROCHECK version 3.5.4, PYMOL version 2.5

For manuscripts utilizing custom algorithms or software that are central to the research but not yet described in published literature, software must be made available to editors and reviewers. We strongly encourage code deposition in a community repository (e.g. GitHub). See the Nature Portfolio guidelines for submitting code & software for further information.

Data

Policy information about availability of data

All manuscripts must include a data availability statement. This statement should provide the following information, where applicable:

- Accession codes, unique identifiers, or web links for publicly available datasets
- A description of any restrictions on data availability
- For clinical datasets or third party data, please ensure that the statement adheres to our policy

The coding sequences of NobZIP77 and NoDGAT2B are deposited in GenBank under MT273120 [https://www.ncbi.nlm.nih.gov/nuccore/MT273120] and KX867957 [https://www.ncbi.nlm.nih.gov/nuccore/KX867957], respectively. Source data are provided with this paper.

Field-specific reporting

Please select the one below that is the best fit for your research. If you are not sure, read the appropriate sections before making your selection.

- [x] Life sciences
- [ ] Behavioural & social sciences
- [ ] Ecological, evolutionary & environmental sciences

For a reference copy of the document with all sections, see nature.com/documents/nr-reporting-summary-flat.pdf

Life sciences study design

All studies must disclose on these points even when the disclosure is negative.

Sample size	No statistical methods were used to predetermine sample sizes. Sample sizes were predetermined on the basis of published studies. All experiments in this study were repeated for at least three times (For EMSA assays under darkness, white light and blue light, experiments were twice-repeated).
Data exclusions	Data analysis in this study was performed on original data directly, without any subjective exclusion.
Replication	Most experiments in this study were repeated for at least three times, with same experimental protocol, followed by same statistical analysis. All attempts at replication were successful and identical. For EMSA assays under darkness, white light and blue light, these experiments were twice-repeated, with same experimental protocol, followed by same statistical analysis. All attempts at replication were successful and identical.
Randomization	The samples were allocated randomly into experimental groups without subjective judgment.
Blinding	The investigators were blinded to group allocation during data collection and analysis.

Reporting for specific materials, systems and methods

We require information from authors about some types of materials, experimental systems and methods used in many studies. Here, indicate whether each material, system or method listed is relevant to your study. If you are not sure if a list item applies to your research, read the appropriate section before selecting a response.

Materials & experimental systems

n/a	Involved in the study	
☐	☒	Antibodies
☐	☒	Eukaryotic cell lines
☒	☐	Palaeontology and archaeology
☒	☐	Animals and other organisms
☒	☐	Human research participants
☒	☐	Clinical data
☒	☐	Dual use research of concern

Methods

n/a	Involved in the study	
☒	☐	ChIP-seq
☒	☐	Flow cytometry
☒	☐	MRI-based neuroimaging

Antibodies

Antibodies used	anti-GFP monoclonal antibody for ChIP-qPCR assays (Abmart M20004, clone7G9, 1/50;Shanghai, China)
Validation	This commercial antibody was validated for use in this system noting their validation statements on the manufacturer's website.

Eukaryotic cell lines

Policy information about cell lines

Cell line source(s)	Nannochloropsis oceanica IMET1 from Single-Cell Center of Qingdao Institute of BioEnergy and Bioprocess Technology
Authentication	Nannochloropsis oceanica IMET1 was confirmed with NGS.
Mycoplasma contamination	Not applicable.
Commonly misidentified lines (See ICLAC register)	No misidentified lines in this study.
